# Negative velocity fluctuations and non-equilibrium fluctuation relation for a driven high critical current vortex state

**DOI:** 10.1038/s41598-017-05191-6

**Published:** 2017-07-17

**Authors:** Biplab Bag, Gorky Shaw, S. S. Banerjee, Sayantan Majumdar, A. K. Sood, A. K. Grover

**Affiliations:** 1 Department of Physics, Indian Institute of Technology, Kanpur, 208016 India; 2 Department of Physics, Indian Institute of Science, Bengaluru, 560012 India; 3 Department of Physics, Panjab University, Chandigarh, 160014 India; 4 Experimental Physics of Nanostructured Materials, Q-MAT, CESAM, Universitè de Liège, Sart Tilman, B-4000 Belgium; 5 James Franck Institute, The University of Chicago, Chicago, Illinois 60637 USA; 6 Department of Condensed Matter Physics and Materials Science, Tata Institute of Fundamental Researc, Mumbai, 400005 India

## Abstract

Under the influence of a constant drive the moving vortex state in 2H-NbS_2_ superconductor exhibits a negative differential resistance (NDR) transition from a steady flow to an immobile state. This state possesses a high depinning current threshold (${{\boldsymbol{I}}}_{{\boldsymbol{c}}}^{{\boldsymbol{h}}}$) with unconventional depinning characteristics. At currents well above ${{\boldsymbol{I}}}_{{\boldsymbol{c}}}^{{\boldsymbol{h}}}$, the moving vortex state exhibits a multimodal velocity distribution which is characteristic of vortex flow instabilities in the NDR regime. However at lower currents which are just above ${{\boldsymbol{I}}}_{{\boldsymbol{c}}}^{{\boldsymbol{h}}}$, the velocity distribution is non-Gaussian with a tail extending to significant negative velocity values. These unusual negative velocity events correspond to vortices drifting opposite to the driving force direction. We show that this distribution obeys the Gallavotti-Cohen Non-Equilibrium Fluctuation Relation (GC-NEFR). Just above ${{\boldsymbol{I}}}_{{\boldsymbol{c}}}^{{\boldsymbol{h}}}$, we also find a high vortex density fluctuating driven state not obeying the conventional GC-NEFR. The GC-NEFR analysis provides a measure of an effective energy scale (*E*_*eff*_) associated with the driven vortex state. The *E*_*eff*_ corresponds to the average energy dissipated by the fluctuating vortex state above ${{\boldsymbol{I}}}_{{\boldsymbol{c}}}^{{\boldsymbol{h}}}$. We propose the high *E*_*eff*_ value corresponds to the onset of high energy dynamic instabilities in this driven vortex state just above ${{\boldsymbol{I}}}_{{\boldsymbol{c}}}^{{\boldsymbol{h}}}$.

## Introduction

Periodic elastic medium of vortices in type II superconductors driven through a random pinning environment, is a powerful prototype for studying non-equilibrium (NE) systems^1–3^. The vortex number density, $n=B/{\phi }_{0}$ (and hence inter-vortex spacing ${a}_{0}\propto {n}^{-1/2}$) is conveniently and continuously varied by changing the magnetic field (*B*), *φ*_0_ is the magnetic flux quanta carried by each vortex. The driving Lorentz force (${\vec{F}}_{L}=\vec{I}\times \vec{B}$) acting on a vortex array is easily changed by the current (*I*) injected into the superconductor. For an elastically pinned vortex array, beyond a critical current threshold (*I*_*c*_), *F*_*L*_ exceeds the pinning force and vortices begin moving. Investigations reveal that the vortex depinning is either a homogenous (elastic depinning) or an inhomogeneous (plastic﻿)﻿ transition^4–16^. Usually a disordered vortex lattice with high *I*_*c*_ depins plastically, forming channels of vortices flowing around islands of strongly pinned vortices (which depin at higher drives). Usually, the application of large driving current anneals a high *I*_*c*_ state into a relatively well ordered low *I*_*c*_ vortex state^10^. However studies in YNi_2_B_2_C crystals^17^ and melt spun Fe_x_Ni_1−x_Zr_2_ samples^18^ have shown that at large driving currents the driven vortex state can also enter a high *I*_*c*_ state. A recent study^19^ in a single crystal of 2H-NbS_2_ shows a drive induced abrupt transformation from a moving to an immobile vortex state with a high critical current, ${I}_{c}^{h}$. Earlier simulation and experimental studies in high *T*_*c*_ superconductors on negative differential resistance (NDR) transitions have shown that at large drives, dynamical vortex flow instabilities can produce a drop in vortex velocity^20–27^.

In this paper using current (*I*) – voltage (*V*) and voltage time series measurements we investigate an abrupt transition from a free flow to an immobile vortex state in three different single crystals of 2H-NbS_2_ with different pinning strengths. The immobile state has a high *I*_*c*_ (${I}_{c}^{h}$) with unconventional depinning properties: The value and behavior of ${I}_{c}^{h}$ are shown to be unaffected by variations in, the amount of order present in the underlying static vortex matter, the pinning strength in the sample and the vortex density. Above ${I}_{c}^{h}$, the depinned state exhibits large velocity fluctuations. At $I\gg {I}_{c}^{h}$, there is an abrupt transition in *I-V* from a state with large fluctuations to a regime with relatively low fluctuations. Close to this high drive regime ($I\gg {I}_{c}^{h}$) the vortex velocity distribution is multimodal, which is characteristic of vortex flow instabilities associated with an NDR regime. At lower *I* (just above ${I}_{c}^{h}$), the fluctuating state exhibits unusual negative velocity events associated with vortices drifting opposite to the drive direction. In this regime the velocity distribution is non-Gaussian with a significant negative velocity tail and the distribution obeys the Gallavotti-Cohen Non-Equilibrium Fluctuation Relation^28–36^ (GC-NEFR). The GC-NEFR analysis provides a measure of an effective energy scale (*E*_*eff*_) and we also determine the large deviation function (LDF) associated with these unusual fluctuation events. The *E*_*eff*_ corresponds to the average energy dissipated by the fluctuating vortex state above ${I}_{c}^{h}$. We propose the high *E*_*eff*_ value corresponds to the onset of dynamic instabilities in this driven vortex state near ${I}_{c}^{h}$, ﻿﻿﻿with significant﻿ly large fluctuations in the energy dissipated.

## Experimental details

We study the driven vortex state in three single crystals of 2H-NbS_2_ (*T*_*c*_ = 5.8 K) superconductor using four probe electrical transport measurements. Henceforth the crystals will be labeled as A1 (2.0 × 1.0 × 0.045 mm^3^), A2 (0.9 × 0.9 × 0.045 mm^3^) and A3 (1.3 × 0.8 × 0.05 mm^3^) samples (where A2 is the crystal of ref. 19). The dc magnetic field was applied parallel to the crystallographic *c*-axis of the single crystals and the current was applied along the *ab*-crystal basal plane. The bulk dc magnetization response of the samples was measured with a SQUID magnetometer (Quantum Design Inc., USA). The residual resistivity ratio’s (RRR = *R*(300 K)/*R*(10 K)) of samples A1, A2 and A3 were 25, 35, and 39 respectively. Note the RRR values of A2 and A3 samples are not significantly different, hence often for the sake of clarity we shall compare data for A1 and A2 samples. We define *I*_*c*_ as the current beyond which the voltage, *V* ≥ 1 μV. For sample A1, we estimate the mean vortex velocity (v) using *V* = *B*v*d*, where *d* *=* 0.53 ± 0.03 mm is the mean separation between the voltage contacts, for eg., *V* = 100 μV at *B* *=* 0.4 T gives v ~ 50 cm/s. Note the protocol for voltage time series, *V*(*t*), measurement has been discussed in the methods section. For all our measurements, we ensured the sample temperature (*T*) was stable to within 5 mK.

## Results

### Bulk Magnetization data and the static vortex phase

**Figure 1:**
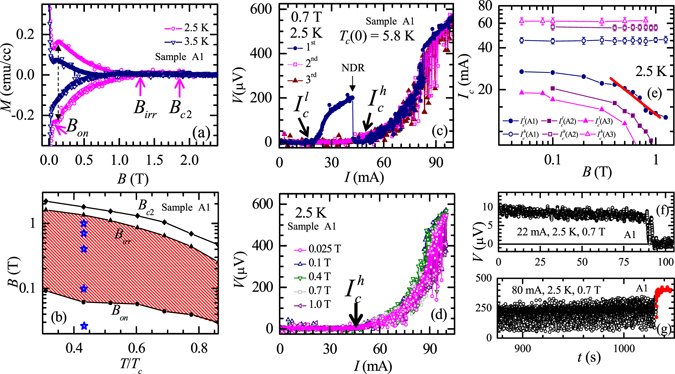
The bulk magnetization data, static vortex phase diagram and electric transport characteristics in 2H-NbS_2_. (**a**) Variation of bulk isothermal magnetization (*M*) response with magnetic field *B* (*B//c*) at 2.5 K and 3.5 K for sample A1. The magnetization hysteresis loop shows the second magnetization peak (SMP) anomaly. The vertical double arrow head line represents location of the maximum increase in *ΔM* at the peak of the SMP anomaly. The characteristics fields *B*_*on*_, *B*_*irr*_ and *B*_*c*2_ (see text for details) are marked with arrows. (**b**) *B*(*T/T*_*c*_) phase diagram for the static vortex state in sample A1 showing variation of *B*_*on*_, *B*_*irr*_ and *B*_*c*2_ with *T/T*_*c*_. The shaded region identifies a partially disordered vortex state whereas the region below *B*_*on*_ represents ordered vortex state. The region between *B*_*irr*_ and *B*_*c*2_ represents a reversible vortex phase. The blue stars represent the *B*,*T* values at which we have measured the *I-V* characteristics in Fig. 1(d). (**c**) The *I-V* response at 2.5 K and 0.7 T for the 1^st^ (blue, filled circles), 2^nd^ (pink, empty square), and 3^rd^ (brown, triangle) runs (see text for details) in sample A1. The magnetic field of 0.7 T was reached by ramping the field at 0.03 T/minute. The *I-V* for the 1^st^ run shows an NDR transition at *I* ~ 40 mA and there after the vortex matter falls to a high *I*_*c*_ state. The ${I}_{c}^{l}$ and ${I}_{c}^{h}$ are marked for the 1^st^ run. (**d**) *I-V* response measured at 2.5 K and different *B* (the locations of *B* are indicated by blue stars in Fig. 1(b)) for sample A1 showing depinning from the high *I*_*c*_ state at ${I}_{c}^{h}$ with large *V*-fluctuations. (**e**) Variations of ${I}_{c}^{l}$ (closed symbols) and ${I}_{c}^{h}$ (open symbols) with *B* at 2.5 K in log-log scale for sample A1 (circle), A2 (square) and A3 (triangle). The red solid line is a fit, ${I}_{c}^{l}\propto \,1/{B}^{\alpha }$ with α ~ 0.6 for sample A1. (**f**) Voltage as a function of time, *V*(*t*) data captured at 0.7 T, 2.5 K with *I = *22 mA, showing transition to a high *I*_*c*_ state after 90 s. (**g**) *V*(*t*) data (only last 150 s data) at 0.7 T, 2.5 K with *I = *80 mA showing transition from a fluctuating (black points) to steady free flow (red points) response after 1025 s.

In Fig. 1(a) we show the bulk magnetization hysteresis loops, *M*(*B*), measured at 2.5 K and 3.5 K for sample A1. At 2.5 K, Fig. 1(a) shows an increase in the width (Δ*M*) of the hysteresis loop (indicated by the dashed vertical (black) line, where Δ*M* becomes maximum) due to a second magnetization peak (SMP) like anomaly^37–40^. The *B* location for onset of the SMP anomaly at 2.5 K is marked as *B*_*on*_ (~0.06 T). The hysteresis loop at 3.5 K (cf. Fig. 1(a)) also shows similar qualitative features. From the *M*(*B*) loops, we also identify the upper critical field, *B*_*c*2_(*T*) (from the onset of diamagnetic response) as well as the irreversibility field, *B*_*irr*_(*T*) (above which the *M*(*B*) is reversible) (see Fig. 1(a)). For sample A1, the *T*-dependence of *B*_*on*_, *B*_*irr*_ and *B*_*c*2_ are summarized through the *B*(*T*/*T*_*c*_) phase diagram in Fig. 1(b). The width of the magnetization hysteresis loop, i.e., Δ*M* (∝*J*_*c*_) increases for *B* > *B*_*on*_(*T*) (see Fig. 1(a)), i.e., across the shaded SMP region in Fig. 1(b) (see also Supplementary- section I, showing enhancement in *J*_*c*_ above *B*_*on*_(*T*)). STM imaging of the vortex state in 2H-NbSe_2_^41^ has already shown that the SMP related increase in *J*_*c*_ (or increased vortex pinning) is associated with a proliferation of topological defects destroying long range order in the vortex lattice. The region below *B*_*on*_(*T*) in Fig. 1(b) is a weak collectively pinned (relatively ordered) vortex solid while the shaded region between *B*_*on*_ and *B*_*irr*_ is relatively more strongly pinned (partially ordered) vortex state. Note that a similar phase diagram has been reported in other samples of 2H-NbS_2_^42^.

### Transport characteristics of drive-induced high *I*_*c*_ state

Figure 1(c) shows the *I-V* curve for a vortex state prepared by zero field cooling (ZFC) the sample A1 at 2.5 K and 0.7 T. From Fig. 1(b) note that the static vortex matter at 0.7 T and 2.5 K is well inside the shaded region above *B*_*on*_(*T*). Figure 1(c) shows that in the first (Forward) *I-V* run (blue circles), after depinning from the static vortex state at ${I}_{c}^{l}$ (2.5 K, 0.7 T) ~ 17.5 mA, the driven vortex state enters a linear flux flow (FF) *I*-*V* regime at *I* > 30 mA. In this linear regime we observe a sharp drop in *V* below the noise floor near 40 mA (marked as an NDR transition (to be discussed later)). Here the vortex state is in an immobile state. In Fig. 1(c) we see that a finite measurable voltage appears only at *I* ≥ ${I}_{c}^{h}$ *~* 45 mA and depinning commences with a highly fluctuating *V-*response, which is unlike the nature of depinning seen at ${I}_{c}^{l}$. In high drive regime between 80–90 mA range, the fluctuations in the *V* abruptly decrease and the *I-V* becomes linear. Without modifying *B* and *T*, we measure *V* while decreasing *I* from 100 mA to zero (viz., the 2^nd^ reverse *I-V* run, pink squares in Fig. 1(c)). From this *I-V* we see the vortex state repins at *I* = ${I}_{c}^{h}$ and not ${I}_{c}^{l}$. This is unlike conventional behaviour where a low *I*_*c*_ state is usually achieved while returning from large drives^10^. For the 3^rd^ forward run (Fig. 1(c), brown triangles), viz., measure *V* while increasing *I* from 0 to 100 mA (*B* and *T* unaltered), we observe the driven vortex state depins only above ${I}_{c}^{h}$ with a fluctuating *V*-response similar to the 1^st^ *I-V* run. Thus the low ${I}_{c}^{l}$ value is accessed only in the first *I-V* run. Beyond the abrupt drop in *V*, always the high ${I}_{c}^{h}$ state is accessed in the *I-V*. We show a similar transformation to the high critical current state using *V*(*t*) measurement at a constant drive in Fig. 1(f) for sample A1. Figure 1(f) shows that after 90 s of switching on 22 mA ($> {I}_{c}^{l}(2.5\,{\rm{K}},0.7\,{\rm{T}}) \sim 17.5$ mA), the mean voltage drops from an almost steady value of 10 µV to below the noise floor i.e., the moving vortex state stops after 90 s. A critical current measurement (*I-V*) of this immobile state (reached via *V*(*t*)) reveals it has a high *I*_*c*_, viz., ${I}_{c}^{h}$.

Using the procedure in Fig. 1(c), in Fig. 1(d) we show depinning (viz., the 3^rd^ *I-V* run like that Fig. 1(c)) from identical ${I}_{c}^{h}$ values at different *B* at 2.5 K. Figure 1(e) summarizes the *B*-dependence of ${I}_{c}^{l}$ and ${I}_{c}^{h}$ for samples A1, A2 and A3 on a log-log scale. Figure 1(e) shows that above *B* ~ 0.5 T, ${I}_{c}^{l}(B)$ behavior, for all samples, obey ${I}_{c}^{l}\propto 1/{B}^{\alpha }$, with α = 0.6 ± 0.1, while ${I}_{c}^{h}(B)$ has a completely different behavior, viz., ${I}_{c}^{h}$ is almost constant and independent of *B*. The ${I}_{c}^{l}\propto 1/{B}^{\alpha }$ behavior is associated with a decrease in effective pinning of a weakly pinned vortex lattice due to enhanced rigidity of the lattice at higher *B* ^9, 11, 43^. Using intrinsic parameters of 2H-NbS_2_^42^ and the lower critical current﻿ density, ${J}_{c}^{l}$ ~ 4.4 × 10^5^ A.m^−2^ (20 mA) for A1 we estimate^44^ the radial correlation length *R*_*c*_ in the weakly pinned vortex state is of the order of 10*a*_*0*_ for the static vortex state. Similarly the high ${I}_{c}^{h}$ corresponds to *R*_*c*_ */a*_*0*_ of the order of 1, viz., a disordered vortex configuration. Here we would like to mention that the low to high *I*_*c*_ transformation in our sample isn’t because of a Peak Effect (PE) phenomenon^8, 9^. The PE phenomenon occurs close to the upper critical field of a superconductor where the *I*_*c*_ is found to increase non-monotonically with increasing *B* or *T* (see PE location in *B-T* vortex phase diagram for 2H-NbS_2_ in ref. 42). Note Fig. 1(e) shows that, irrespective of whether *B* values are near or far away from the upper critical field, the critical current transforms from a low to high ${I}_{c}^{h}$ state. Furthermore, unlike the behavior of *I*_*c*_ in the PE regime which increases with *B* until the peak of PE phenomena, the ${I}_{c}^{h}$ is independent of *B* (see Fig. 1(d) and (e)). Also note that while ${I}_{c}^{l}$ shows the expected decrease with increasing RRR value (RRR: A1 > A2 > A3) i.e., a sample with lower RRR value has higher intrinsic pinning, the ${I}_{c}^{h}(B)$ behavior is not correlated with the RRR value of the sample. We have also shown that unlike depinning above ${I}_{c}^{l}$, there are unusually large *V* fluctuations found above ${I}_{c}^{h}$ (with peculiar negative voltage fluctuations, shown later). From all of the above it appears that depinning above ${I}_{c}^{h}$ is unconventional. (Another distinguishing feature of depinning above ${I}_{c}^{h}$ is the observation of diverging transient timescales (see ref. 19 and Supplementary-section IV)). We now investigate features near ${I}_{c}^{h}$ closely.

### Behavior of *I-V* and the probability distribution of *V*(*t*) at different drives

**Figure 2:**
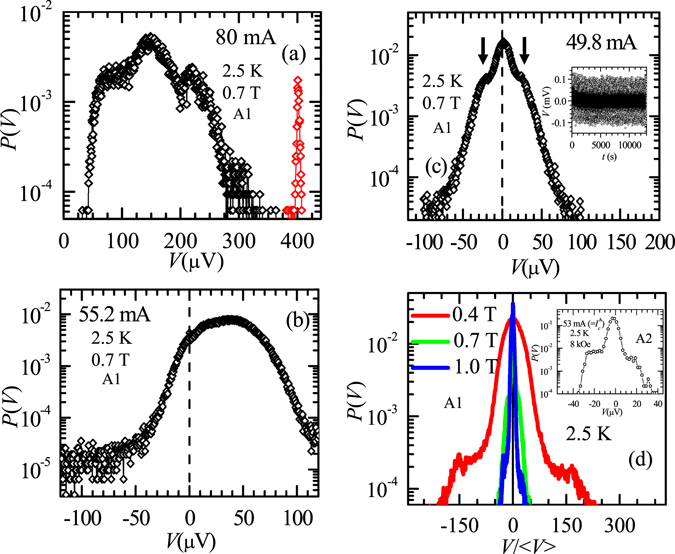
The probability distribution of output voltage. The probability distribution *P*(*V*) estimated from *V*(*t*) measured for sample A1 at 2.5 K and 0.7 T with *I* = (**a**) 80 mA, (**b**) 55.2 mA and (**c**) 49.8 mA. The inset of (**c**) presents the *V*(*t*) series measured at 2.5 K, 0.7 T with *I* = 49.8 mA. The vertical dashed straight lines in (**b**,**c**) identify *V* = 0 μV line. (**d**) *P*(*V*) vs *V*/〈*V*〉 for sample A1 at 2.5 K and *I* = 49.8 mA for different *B*: 0.4, 0.7 and 1.0 T. Inset shows *P*(*V*) for sample A2 estimated from *V*(*t*) at 2.5 K, 8 kOe with *I* ~ 53 mA $( \sim {I}_{c}^{h})$.

Recall the *I-V* in Fig. 1(c) shows an abrupt voltage drop at 40 mA from *V* = 200 µV to *V* < 1 μV (corresponding to a drop in v from 54 cm/s to about zero). This drop is similar to the voltage drop in *I-V* found due to vortex flow instabilities associated with an NDR transition^20–27^. Using the normal state resistivity of our samples *ρ*_*n*_ ~ 60 μΩ-cm, the Bardeen-Stephen flux flow resistivity is estimated to be, ${\rho }_{f}={\rho }_{n}\frac{B}{{B}_{c2}} \sim 16.8$ μΩ-cm (we use *B* = 0.7 T and *B*_c2_(2.5 K) ~ 2.5 T). In Fig. 1(c), at 40 mA (onset of transition in *I-V*) the value of resistivity ρ* ~ 42.5 μΩ-cm ~ 2.5 *ρ*_*f*_, which is similar to the criteria for an NDR transition (viz., ρ* ~ 2*ρ*_*f*_)^22^, where abrupt changes in vortex viscosity at high velocities produce vortex flow instabilities and a drop in vortex velocity^22^. However compared to NDR transition where above the transition the vortex velocity is low but measurable, in our 2H-NbS_2_ samples above the NDR transition the vortex-velocity abruptly drops below measurable limits into a state with high ${I}_{c}^{h}$, which has unconventional depinning characteristics mentioned earlier. Recall, we had already discussed that at a current in 80 to 90 mA range, the noisy *I-V* in Fig. 1(c) jumps to a linear *I-V* which is relatively less noisy. In Fig. 1(g) we explore closely the *V*(*t*) response in this high current regime, viz., at 80 mA, in sample A1. In Fig. 1(g), we observe large voltage fluctuations (~200 μV) persisting for about 1025 s (black open circles), after which the voltage abruptly transforms into uniform FF regime with a steady voltage (red data points). Similar features in *V*(*t*) for *I* ≫ ${I}_{c}^{h}$ have been shown earlier for A2^19^ and similar features also exist in A3 (data not shown). For sample A1, the probability distribution of voltages, *P*(*V*), determined from the *V*(*t*) data (which have approximately 5 × 10^5^ data points), captured at different *I* > ${I}_{c}^{h}$ are displayed in Fig. 2(a–d). For 80 mA (corresponding *V*(*t*) is shown in Fig. 1(g)), the shape of *P*(*V*) in Fig. 2(a) suggests a multimodal nature of vortex velocity distribution. The distribution has a broad maximum at ~150 μV (v ~ 40 cm/s), corresponding to large voltage fluctuations found in *V*(*t*) below 1025 s (black open circles in Fig. 1(g)) and another narrower (red) maximum (corresponds to red data points in *V*(*t*) in Fig. 1(g)) at 400 μV (v ~ 107 cm/s). The multimodal *P*(*V*) at 80 mA implies a transformation from a flow with large velocity fluctuations below 1025 s (broad *P*(*V*)) into a FF state with a relatively uniform velocity distribution (narrow *P*(*V*)). Theories suggest N or S shaped features in *I-V* signifying vortex flow instability in NDR regime^26, 27^. It is proposed that in this instability regime flow switches between channel like flow with a distribution of vortex flow velocities^24–27^ to a uniform flow state. In the *I-V* regime with N or S-type instability hysteresis is also expected. In Fig. 2(a) the multimodal vortex velocity distribution at high drives we believe is associated with the above dynamical instability in the NDR regime. Figure 1(c) (and Supplementary-section II for a zoomed view) also hints of a hysteretic *I-V* behavior i﻿n this NDR regime although it is masked by the large *V* fluctuations. Thus, from our *I-V* and *V*(*t*) measurements we see an NDR regime begins from 40 mA and extends upto high *I* values. However as discussed earlier the state acquired just above the NDR transition at 40 mA exhibits unconventional features. In Fig. 2(b–d) we explore the nature of *P*(*V*) distribution as *I* → ${I}_{c}^{h}$.

On reducing *I* to 55.2 mA, the *P*(*V*) distribution (Fig. 2(b)) changes from a multimodal to a non-Gaussian distribution, with a maximum at ~35 μV. While the velocity distribution is still quite broad at 55.2 mA, a noteworthy feature is the tail of the distribution extending to significantly large negative voltage (velocity) values. The negative voltage (velocity) events correspond to vortices drifting opposite to the driving force direction. Inset of Fig. 2(c) shows the *V*(*t*) data measured at 2.5 K and 0.7 T with *I* = 49.8 mA and the main panel of Fig. 2(c) shows *P*(*V*) becomes non-Gaussian with a significant negative velocity tail, suggesting the probability for observing negative voltage events becomes larger as *I* approaches closer to ${I}_{c}^{h}$. For *I* = 49.8 mA (i.e., close to ${I}_{c}^{h}$ ~ 45 mA), *P*(*V*) in Fig. 2(c) shows two shoulders peaked at *V* ~ ±22 µV (cf. arrows in Fig. 2(c)) and an overall maximum at 〈*V*〉 ~ 2 µV. This *P*(*V*) implies that the depinned vortex state while on an average drifts in the direction of drive with 〈*V*〉 ~ 2 μV, it also exhibits large forward and backward motion (w.r.t drive direction), resulting in shoulders at *V* ~ +22 μV and −22 µV, respectively. For sample A2 also, we have observed a *P*(*V*) distribution similar to Fig. 2(c) near ${I}_{c}^{h}$ (cf. Fig. 2(d) inset). The significant values of *P*(*V*) for negative voltage events in the inset of Fig. 2(d) indicate unusual backward flow of vortices w.r.t the drive direction. Note that negative velocity fluctuations haven’t been reported earlier close to any conventional vortex depinning regimes^4–14, 20–27^. However, driving a colloidal system out of its jammed state shows unusual negative and positive fluctuations in shear rate^28^ which are analogous to the negative and positive vortex velocity fluctuations we observe near ${I}_{c}^{h}$.

One expects that vortex velocity fluctuations may get modified by changing repulsive interactions at different vortex densities. Hence, it is worthwhile investigating the change in the behavior of *P*(*V*) with *B*. Figure 2(d) shows *P*(*V*) versus $V/\langle V\rangle$ measured close to *I* = 49.8 mA (2.5 K) for different *B* in sample A1. The $V/\langle V\rangle$ is equivalent to the spread in vortex velocity. While the shape of the distribution at different *B* are similar, the distribution is broad for low vortex density at *B* = 0.4 T which narrows down significantly for higher vortex densities at *B* = 0.7 T and 1.0 T (note for different *B*, 〈*V*〉 is between 2–4 µV). The tails of *P*(*V*) which are spread over $V/\langle V\rangle$ ~ ±180 at 0.4 T, consistently decrease to about ±45 at 0.7 T and 1.0 T under identical conditions of temperature and current. The above systematic behavior of *P*(*V*) with *B*, the stability of *T* and other measurements [see Supplementary, section III] confirm that the observed fluctuations aren’t artifacts or random electronic noise.

### The shape of *P*(*V*) and the GC-NEFR close to ${{\boldsymbol{I}}}_{{\boldsymbol{c}}}^{{\boldsymbol{h}}}$

Within a finite time window of observation of a dissipating driven system, for example a driven collection of particles with random inter-particle collisions, while most of the particles will be seen to drift in the drive direction there also exist events associated with particles drifting opposite to the drive. The energy exchanges in non-equilibrium systems with a positive mean velocity under steady state flow conditions are governed by GC-NEFR^31–36^. GC-NEFR states the probability of power generating events (events associated with particles drifting opposite to drive, or negative velocity events) is exponentially small compared to power consuming events (events associated with particles drifting in the direction of the drive, or positive velocity events). The GC-NEFR has been experimentally verified in various driven systems like, dragged colloidal particles in an optical trap, electrical circuits and jammed state in sheared micellar gel^28, 45–49^. We analyse the non-Gaussian *P*(*V*) distribution with negative velocity tail near ${I}_{c}^{h}$ (see Fig. 2(d)), in terms of *P*(*W*_*τ*_) and GC-NEFR, where the power consumed or produced in a finite time interval is *W*_*τ*_. From the *V*(*t*) data at *I* = 49.8 mA, a series of *W*_*τ*_ values are calculated by breaking up the *V*(*t*) data into bins of width *τ* [for details see methods section], viz.,

1 $${W}_{\tau }={s}_{\tau }/\langle s(t)\rangle =\frac{\frac{1}{\tau }{\int }_{t}^{t+\tau }IV(t^{\prime} )dt^{\prime} }{I\langle V\rangle }.$$

**Figure 3:**
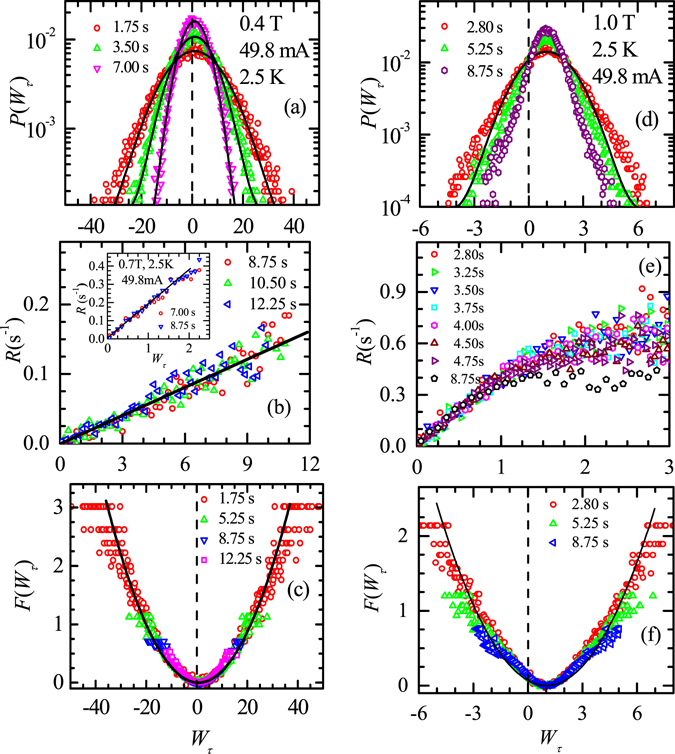
Validity of GC-NEFR and estimation of LDF. (**a**) Probability distribution *P*(*W*_*τ*_) vs *W*_*τ*_ at 0.4 T, 2.5 K, and 49.8 mA for *τ* = 1.75, 3.50, and 7.00 s. The solid lines are fitted Gaussian’s. (**b**) *R* vs *W*_*τ*_ (estimated from *P*(*W*_*τ*_) in Fig. 3a) for *τ* = 8.75, 10.50, and 12.25 s. Inset shows *R*(*W*_*τ*_) behavior for *τ* = 7.00 and 8.75 s at 0.7 T, 2.5 K, and 49.8 mA data. (**c**) The LDF, *F*(*W*_*τ*_) for *τ* = 1.75, 5.25, 8.75 and 12.25 s (estimated from *P*(*W*_*τ*_) in Fig. 3a). The solid line represents a quadratic fitting to *F*(*W*_*τ*_) data sets. (**d**) *P*(*W*_*τ*_) vs *W*_*τ*_, for *τ* = 2.80, 5.25 and 8.75 s at 1.0 T, 2.5 K and 49.8 mA. The solid line is a Gaussian fit to the data with *τ* = 2.80 s showing a clear deviation. (**e**) The *R*(*W*_*τ*_) behavior for different *τ*’s. Clearly, the *R*(*W*_*τ*_) behavior is not linear. (**f**) The LDF for *τ* = 2.80, 5.25 and 8.75 s. The solid line is the quadratic fit to *F*(*W*_*τ*_) data sets (cf. text for details). The vertical dashed lines in (**a**,**c**,**d**,**f**) identify the *W*_*τ*_ = 0 line.

In terms of *W*_*τ*_, the GC-NEFR is restated as in ref. 28,

2 $$R=\frac{1}{\tau }\,\mathrm{ln}(P(+{W}_{\tau })/P(-{W}_{\tau }))={s}_{\tau }={W}_{\tau }\langle s(t)\rangle $$

with $s(t)=IV(t)/{E}_{eff}$ and *τ* is time window of observation. Due to averaging over *τ*, the shoulders in *P*(*V*) (Fig. 2(d)) are smoothened out in *P*(*W*_*τ*_) in Fig. 3(a and d) for different *τ* at 0.4 T and 1.0 T at 49.8 mA. In *P*(*W*_*τ*_) distribution note the significant probability for observing negative *W*_*τ*_ events (viz., power generating events) representing the unusual negative vortex velocity events or vortex backflow associated with vortices moving against the driving force. The positive voltage, *W*_*τ*_ events are the conventional power consuming dissipating vortex flow trajectories drifting along the drive direction. The scaled linear *R* vs *W*_*τ*_ for different *τ* in Fig. 3(b) (and inset) verifies GC-NEFR (i.e. Eq. 2) for the depinned vortex state at 0.4 T (and 0.7 T), 2.5 K driven with *I* = 49.8 mA. Notice that unlike 0.4 T (Fig. 3(a)) the *P*(*W*_*τ*_) at 1.0 T (Fig. 3(d)) shows deviation from Gaussian fits to *P*(*W*_*τ*_) (see solid line curves in Fig. 3(a,d)). Notably, Fig. 3(e) shows that *R*(*W*_*τ*_) is non-linear and hence GC-NEFR fails at 1.0 T. Note at $I\gg {I}_{c}^{h}$ as *P*(−*W*_*τ*_) is not discernible, GC-NEFR analysis is no longer feasible in these regimes.

### Estimation of the Large Deviation Function

The Large Deviation Function (LDF)^50^ is considered to play an important role (akin to the role of free energy function for equilibrium systems) for studying non-equilibrium systems and NE phase transformation in such systems^51, 52^. For a system out of equilibrium possessing a fluctuating variable *X* (values are fluctuating with time), the probability *P* of observing large values of *X* (say a value *a*) (large corresponds to value of the variable *X* far away from the mean value) within a time interval (*τ*) is,

3 $$P({X}_{\tau \to \infty }=a)\propto {e}^{-\tau F(a)}$$

where *F*(*a*) is the LDF. Note from Eq. 3, $F(a)\propto \,\mathrm{ln}\,[P({X}_{\tau \to \infty }=a)]$ and if *F*(*a*) and its antisymmetric part obeys, $F(a)-F(-a)\propto a$, then this is simple restatement of GC-NEFR^53, 54^. Using *X* = *W*_*τ*_, Eq. 3 is rewritten as ref. 53,

4 $$P({W}_{\tau })={A}_{\tau }\exp (-\tau F({W}_{\tau }))$$

where *A*_*τ*_ is a positive constant. Using the *P*(*W*_*τ*_) data of Fig. 3(a and d) in Eq. 4 we determine the LDF, *F*(*W*_*τ*_) (cf. Fig. 3(c,f) respectively) in the vortex depinning transition regime just above ${I}_{c}^{h}$ (we determine *A*_*τ*_ using the peak value of *P*(*W*_*τ*_)). The solid line through the data in Fig. 3(c) is a fit to *F*(*W*_*τ*_) of the form *b*(*W*_*τ*_ − 〈*W*_*τ*_〉)^2^, where mean 〈*W*_*τ*_〉 ~ 0.67 ± 0.13, *b* = 2.33 (±0.03) × 10^−3^ s^−1^. Note a failure of GC-NEFR at 1.0 T at higher *W*_*τ*_ (cf. Fig. 3(e)) and the deviation from the quadratic behavior at 1.0 T as seen in Fig. 3(f) suggest, a more general form of GC-NEFR may exist.

## Discussion

In Fig. 3(b,e), the slope of *R*(*W*_*τ*_) is given by,

5 $$\langle s(t)\rangle =I\langle V(t)\rangle /{E}_{eff}$$

The GC-NEFR provides an estimate of an effective energy scale (*E*_*eff*_) associated with dissipation in the fluctuating driven vortex state just above ${I}_{c}^{h}$. Using Eq. 5, we estimate *E*_*eff*_ ~ 0.75 (±0.12) μJ, 0.46 (±0.11) μJ and 0.12 (±0.06) μJ at *B* = 0.4 T, 0.7 T and 1.0 T respectively (note at 1.0 T, *E*_*eff*_ is estimated from the initial linear portion of the *R*(*W*_*τ*_) curve in Fig. 3(e)). This *E*_*eff*_ corresponds (order of magnitude) to the energy associated with 2.5 μW of power dissipated by the fluctuations in the driven vortex state above ${I}_{c}^{h}$ over a time interval, *t* ^*w*^ = 0.1 s, where *t* ^*w*^ is the average time for a vortex to drift across the sample width (Note 2.5 μW = 49.8 mA × 50 μV, where from *P*(*V*) in Fig. 2(c) we use 50 μV for estimating of the typical size of large *V* fluctuations (well above 〈*V*〉) and to estima*t*e *t* ^*w*^ close to ${I}_{c}^{h}$ from the mean 〈*V*〉 of *P*(*V*) at 49.8 mA (Fig. 2(c) we estimate 〈v〉 ~ 1 cm.s^−1^ which for our sample width of 1 mm gives *t* ^*w*^ ~ 0.1 s)). It is important to mention here that using GC-NEFR the non-equilibrium energy scale (*E*_*eff*_) has also been determined for colloidal systems driven into a jammed state^28^.

Recall that both experimental and theoretical studies show that vortex velocities drop to a low, non-zero value at the NDR transition^22–24, 26^. However in our experiments we observe that via the NDR transition vortices reach an immobile state with high ${I}_{c}^{h}$. We propose generation of dynamic instabilities in the NDR regime ceases vortex flow completely in our system. This high ${I}_{c}^{h}$ state exhibits unconventional depinning properties, viz., the absence of significant field dependence of ${I}_{c}^{h}$, presence of velocity fluctuations above ${I}_{c}^{h}$ with unusual negative velocity events, the transient time period (*τ*_*h*_) over which velocity fluctuations are sustained exhibits a critical diverging behaviour of the form ${\tau }_{h}\,\propto \,1/{|I-{I}_{c}^{h}|}^{\beta }$ (see Supplementary section IV and ref. 19) and validity of GC-NEFR. In general, a system driven into a non-flowing state is found when the system undergoes transition to a jammed state, for e.g., jamming in sheared colloidal flows^28, 55, 56^ and vortex flows in honeycomb pinning arrays^57, 58^. In these systems the jamming transition exhibits features like (i) significantly enhanced unjamming force^57, 58^, (ii) observation of fluctuations, especially negative fluctuation^28^ near unjamming and (iii) the validity of fluctuation relations^28, 56^. The similarity of these features tempt us to speculate that the high ${I}_{c}^{h}$ vortex state reached via the NDR transition is different from conventional depinning and could be akin to a dynamically generated jammed state.

The fluctuating flowing vortex state above ${I}_{c}^{h}$ is characterized with an effective energy scale *E*_*eff*_. We recall here the concept of a shaking temperature^59^, *T*_*sh*_ (∝1/v). The shaking temperature has been used to describe velocity fluctuations generated in a moving vortex lattice as they are driven past pinning centers. Although qualitatively *T*_*sh*_ is a useful concept for describing dynamic vortex phase transitions^2, 60–62^, quantitative estimates of *T*_*sh*_ (or the associated energy scale) aren’t well established in literature. Therefore, we cannot establish any connection between *E*_*eff*_ calculated above and *T*_*sh*_. Another relevant energy scale associated with the depinning phenomenon is collective vortex activation energy scale, *U*_*pin*_. The typical values of *U*_*pin*_ ranges from 10^−16^ to 10^−14^ μJ (10 *k*_*B*_ to 10^3^ *k*_*B*_ range^1, 63, 64^). The effective dissipation energy scale, *E*_*eff*_ is much larger than *U*_*pin*_. It may be noted that *E*_*eff*_ is also much larger than the energy of the thermal bath (*k*_*B*_ *T* ~ 2.5 *k*_*B*_). The above suggests that neither thermal fluctuations nor conventional pinning energies can be a source of the large vortex velocity fluctuations found above ${I}_{c}^{h}$. Recent work on the behavior of out of equilibrium systems suggests the existence of definite relationship between dissipation and fluctuations, as it has been found that dissipation not only regulates fluctuations but also puts upper bounds on the large deviation function which governs the statistics of large fluctuation in such driven systems^65^. We propose that *E*_*eff*_ (measured through GC -NEFR analysis) corresponds to the dissipation generated in the unstable NDR flow regime above ${I}_{c}^{h}$ which agitates the vortex flow significantly thereby producing large velocity fluctuations. In this flow regime with dynamically generated instabilities it is quite likely there exist situations where vortices move opposite to the drive, viz., negative velocity events. We have shown in this paper that, the distribution of these velocity fluctuations close to ${I}_{c}^{h}$ obeys GC-NEFR and we determine the large deviation function associated with the statistics of large fluctuation (see Fig. 3).

In conclusion, the NDR (or the jamming) transition in vortex matter is a useful prototype for studying non-equilibrium phase transition using tools like GC-NEFR and LDF. The work paves the way for future detailed theoretical and experimental investigations into unusual non-equilibrium vortex matter phases and transitions between them.

## Methods

### Protocol of *V*(*t*) measurement

After preparing the static vortex state at a certain *B* and *T*, we drive the vortex matter by sending dc current in the sample to reach the high *I*_*c*_ state in the driven vortex matter (similar to Fig. 1(c)). In this state after switching on a fixed current and settling the voltage to a uniform mean value, the voltage time series *V*(*t*) is measured with a time resolution of 35 ms. During all our measurements, we have ensured that the sample temperature was stable within 5 mK.

### Estimation of *W*_*τ*_ series

The time-series of *W*_*τ*_ values are estimated by breaking up *V*(*t*) data into bins of width *τ* using Eq. 1. For better statistical accuracy, we have used overlapping bins. We also have checked that the probability distribution of *W*_*τ*_, *P*(*W*_*τ*_), is same for both overlapping and non-overlapping bins, however the statistical accuracy is much higher in overlapping case. To avoid any correlation between the bins, we have shifted the center of each bin from the previous one by a time difference which is greater than the correlation time associated with the *V*(*t*). The correlation in *V*(*t*) is determined using ${C}(t)=\langle V(t)V(t^{\prime} )\rangle$ which for a time series measurement estimates the amount of correlation of parameter at *t* with its past and future values. We find that close to ${I}_{c}^{h}$, the correlation time, *t*_c_ = 0.28 s. Hence for independent sampling, we have shifted the bins by a time scale >0.28 s for our analysis.

## Electronic supplementary material

supplementary information
