# Supplementary material for: Negative velocity fluctuations and non-equilibrium fluctuation relation for a driven high critical current vortex state
Source: Sci Rep. 2017 Jul 17;7:5531. doi: 10.1038/s41598-017-05191-6 (PMC5514132; doi:10.1038/s41598-017-05191-6)
Supplement: Supplementary file 1 — supplementary information [file 41598_2017_5191_MOESM1_ESM.pdf]

Supplementary Information

Negative velocity fluctuations and non-equilibrium fluctuation relation for a driven high critical current vortex state

Biplab Bag[1], Gorky Shaw[1,4], S. S. Banerjee[1,*], Sayantan Majumdar[2,5], A. K. Sood[2,+] and A. K. Grover[3,#]

[1]*Department of Physics, Indian Institute of Technology, Kanpur-208016, India*

[2]*Department of Physics, Indian Institute of Science, Bengaluru 560012, India*

[3]*Department of Condensed Matter Physics and Materials Science, Tata Institute of Fundamental Research, Mumbai 400005, India*

[4]*Present address: Université de Liège, Département de Physique, Sart Tilman, B-4000, Belgium*

[5]*Present address: James Franck Institute, The University of Chicago, Chicago, Illinois 60637, USA.*

Section I: Disordered vortex phase in SMP region

In Fig. 1(a) in the manuscript, we have already shown an enhancement in the width of the bulk magnetization hysteresis loop above $B_{on}(T)$ in the so-called SMP regime (see text). Using the width (ΔM) of the magnetization hysteresis loop, we estimate the bulk critical current density (J_c) for sample A1 using $J_c(B) = \frac{2\Delta M(B)}{w(1-\frac{w}{3l})}$ (in Amp/m^2) [1] where l and w are sample dimensions with $l > w$. In Fig. S1(a), we

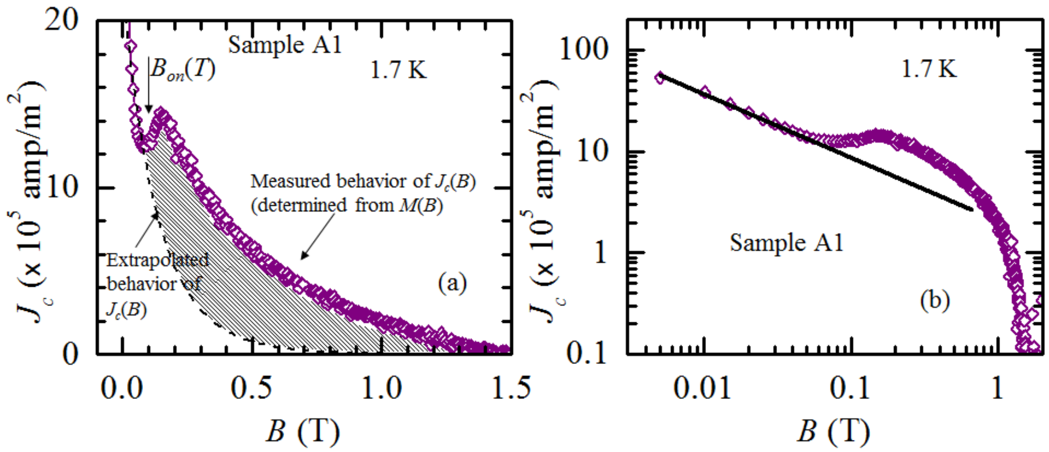

Figure S1: (a) J_c versus B behavior in the SMP region at 1.7 K for sample A1. The dashed line represents the represents the conventional $J_c(B)$ behavior. The shaded region indicates the excess J_c in sample A1 due to enhancement in bulk pinning in the SMP region. (b) the $J_c(B)$ behavior in log-log scale at 1.7 K for sample A1. The thick black line represents the conventional $J_c(B)$ behavior in log-log scale.

plot the $J_c(B)$ behavior of the sample A1 at 1.7 K with B□c . The shaded region in Fig. S1(a) identifies the SMP region. Similar to the $\Delta M(B)$ response in Fig. 1(a), the $J_c(B)$ behavior (Fig. S1(a)) also elucidates an anomalous enhancement in J_c values at $B > B_{on}$. In Fig. S1(a), the dashed line is the $J_c \propto 1/B^{0.5}$ extrapolated behavior which represent the (conventional) monotonic decreasing $J_c(B)$ behavior associated with a weak collectively pinned vortex solid . Note that, within the SMP region i.e., within the shaded

region in Fig. S1(a), the measured $J_c(B)$ values (symbols) remain higher than the $J_c \propto 1/B^{0.5}$ extrapolated behavior (dashed line in Fig. S1(a)). This behavior is a hallmark of enhancement in the bulk pinning associated with SMP which corresponds to relatively a more disordered vortex state at $B > B_{on}(T)$ compared to that below it. The above feature also can be observed in Fig. S1(b) in which the $J_c(B)$ response (at 1.7 K as shown in Fig. S1(a)) is plotted on a log-log scale. The thick black line represents the $J_c(B)$ behavior associated with a weak collectively pinned vortex solid, viz., $J_c \propto 1/B^{0.5}$. The increase in $J_c(B)$ values above the onset of the SMP ($B_{on}(T)$) in our sample is similar to that STM studies in Ref. [2], which shows the increase in pinning across SMP is associated with disordering of the vortex state.

Section II: Hysteresis in *I*-*V* response

In Fig. S2, we replot the *I*-*V* curves shown in Fig. 1(c) for the 1st and 2nd runs (i.e., forward and reverse runs respectively) and in the high current regime (between 50 to 100 mA range). An important feature to observe in Fig. S2 is the hint of finite hysteresis present the forward (1st) and reverse (2nd) *I*-*V* runs in the high current regime ($I > 80$ mA). Here, we would like to mention that although a hysteresis is present in the *I*-*V* data close to $I = 83$ mA, it is masked by the large *V*-fluctuation.

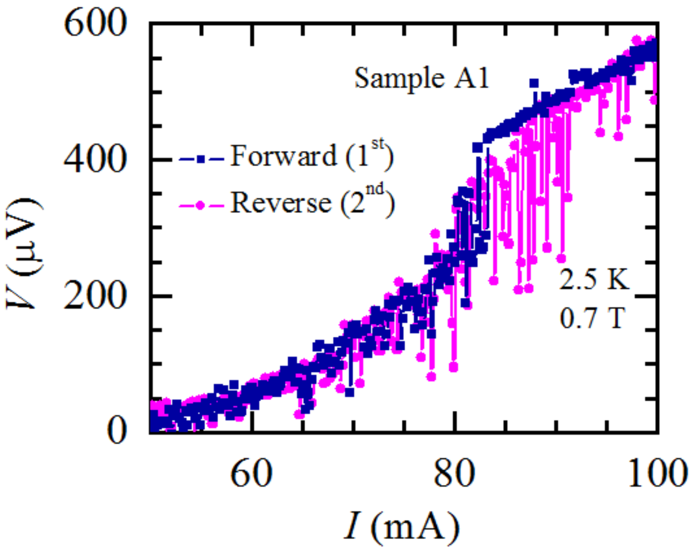

Figure S2: ***I-V*** **characteristics (zoomed-in view of Fig. 1(c) for** $I > I_c^h$ **= 45 mA) of sample A1 at 2.5 K and 0.7 T for forward and reverse (1st and 2nd in Fig. 1(c) respectively) runs.**

Section III: Temperature stability and electronic noise issues.

Along with *V*(*t*), the measured time series of sample temperature fluctuations (for sample A1), *T*(*t*), at 2.5 K are shown in Fig. S3 (the *T*(*t*) traces are shifted artificially for clarity) with *I* = 49.8 mA maintained during the measurement. The temperature fluctuations $\delta T \leq 5$ mK and $\delta T/\langle T\rangle \sim 0.002$, is orders of magnitude smaller than the maximum excursion in $V/\langle V\rangle$ in Fig. 2(d). To discount the possibility that the observed large ±*V* fluctuations arise from random electronic pickup or electronic noise, we find that for *B* = 0 T (no vortex state) at 2.5 K with *I* = 49.8 mA gives $\left|\delta V/V\right| \leq 0.12$ and at 37 K (> T_c) with an applied *B* = 0.7 T, $\left|\delta V/V\right| \leq 0.15$. Both values are orders of magnitude smaller than $\left|\delta V/V\right|$ near depinning from the high I_c state. The above suggests the fluctuations in *V* are intrinsic to the nature of the driven vortex state.

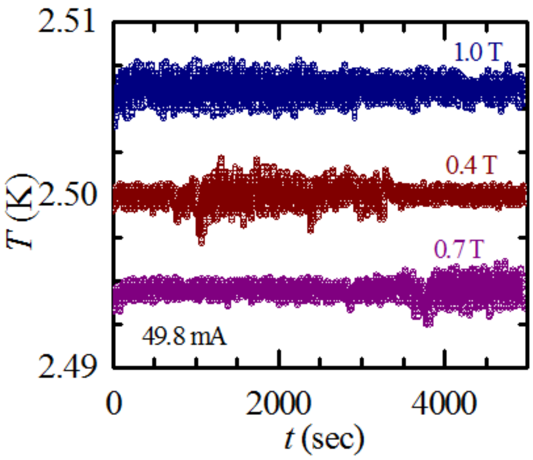

Figure S3: The time series of sample temperature fluctuations, ***T*****(*****t*****), measured along with** ***V*****(*****t*****) at** ***T*** **= 2.5 K and** ***I*** **= 49.8 mA for different** ***B*** **(0.4 T, 0.7 T and 1.0 T) for sample A1. The** ***T*****(*****t*****) traces are shifted artificially for clarity.**

Section IV: Critical like behavior of time scales at $I = I_c^h$.

Studies have shown near plastic depinning Ref. [3] or unstable regimes of vortex flow [4] there are unusual divergences in transient timescales associated with the driven vortex state. It has been shown that, after reaching the high critical current (I_c^h) vortex state reached through the abrupt fall in V in the IV, if a ($V(t)$) is measured on a vortex state driven with a current greater I_c^h then, in this drive state the $V(t)$ shows large fluctuations in V [4]. These fluctuations are sustained for a transient time interval τ_h^f after which the fluctuating vortex state transforms into a free flow state. We identify below in Figs. S4(a) and S4(b) this the transient regime in the sample A1, $\tau_{h,A1}^f$, and in the sample A2, $\tau_{h,A2}^f$. We see in both samples A1 and A2 as $I \to I_c^h$ the transient time τ_h^f becomes large. Figure S4(c) shows the behavior of $\tau_{h,A1}^f$ and $\tau_{h,A2}^f$ versus $\left(\frac{I}{I_c^h} - 1\right)$ for $I > I_c^h$. Figure S4(c) elucidates the diverging nature of τ_h^f as I approaches I_c^h. The dashed curves through the data points are fits to the equation $\tau_h^f \propto (\frac{I}{I_c^h} - 1)^\beta$ where β = -1.40 ± 0.11 and -1.60 ± 0.12 for sample A1 and A2 respectively. This behavior of τ_h^f had been shown for sample A2 in Ref. [4].

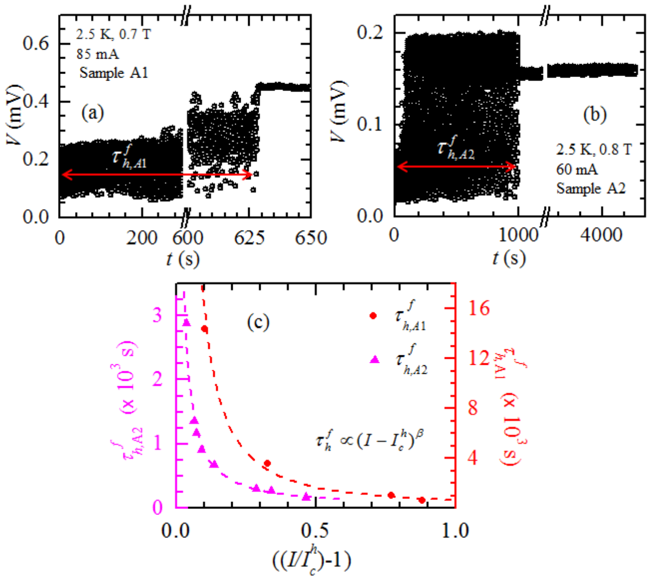

Figure S4: The $V(t)$ response measured at (a) 2.5 K, 0.7 T with I = 85 mA (>> I_c^h = 45 mA) for sample A1 and (b) 2.5 K, 0.8 T with I = 60 mA (>> I_c^h = 56 mA) for sample A2. It shows a highly fluctuating $V(t)$ response which after a transient time of τ_h^f transforms into a free flow state. In (a) and (b) we have identified the τ_h^f values for sample A1 and A2 as $\tau_{h,A1}^f$ (~ 625 s) and $\tau_{h,A2}^f$ (~ 1000 s) respectively. (c) Variation of $\tau_{h,A2}^f$ (on left axis) and $\tau_{h,A1}^f$ (on right axis) against $\left(\frac{I}{I_c^h}-1\right)$ for sample A2 and A1 respectively showing a critical line behavior in the transient time scales around I = I_c^h. The dashed curves are fitting to the curve $\tau_h^f \propto (I-I_c^h)^\beta$ with β = -1.40 and -1.60 for sample A1 and A2 respectively

[1] Bean, C. P. Magnetization of high field superconductors. *Rev. Mod. Phys.* **36**, 31(1964

[2] Zehetmayer, M. How the vortex lattice of a superconductor becomes disordered: a study by scanning tunnelling microscopy. *Sci. Rep* **5**, 9244 (2015).

[3] Okuma, S., Tsugawa, Y. & Motohashi, A. Transition from reversible to irreversible flow: Absorbing and depinning transitions in a sheared-vortex system. *Phys. Rev. B* **83**, 012503 (2011).

[4] Shaw, G. *et al.* Critical behavior at depinning of driven disordered vortex matter in 2H-NbS_2. *Phys. Rev. B* **85**, 174517 (2012).
